# Oral Corticosteroid Relieves Post-COVID-19 Anosmia in a 35-Year-Old Patient

**DOI:** 10.1155/2020/5892047

**Published:** 2020-08-08

**Authors:** Sam K. Touisserkani, Azin Ayatollahi

**Affiliations:** ^1^Private Office, Tehran, Iran; ^2^Tehran University of Medical Sciences, Center for Research and Training in Skin Diseases and Leprosy, Tehran, Iran

## Abstract

Coronavirus disease 2019 (COVID-19) is a highly infectious respiratory illness caused by SARS-CoV-2. Not much is yet known about this new viral disease. In our early encounter with the COVID-19, before the pandemic and during the early days of dealing with this novel viral disease in our country, we saw some cases of anosmia in patients infected by SARS-CoV-2. In many cases, this chemosensitive dysfunction persisted after the negativization of the nasopharyngeal swab. We report effective treatment of anosmia by oral corticosteroid therapy in a patient recovered from COVID-19.

## 1. Introduction

Coronavirus disease 2019 (COVID-19) is a highly infectious respiratory illness caused by SARS-CoV-2. To this date, a range of symptoms from mild symptoms such as fever and dry cough to severe ARDS and death has been reported [1]. Not much is yet known about this new viral disease.

Olfactory dysfunction might be caused after upper respiratory tract infections (URIs). In the case of COVID-19, we saw some cases of anosmia in patients during the course of the disease and mostly after recovery. Herein, we report effective treatment of anosmia by oral corticosteroid therapy in a patient recovered from COVID-19.

## 2. Case Report

**Figure 1:**
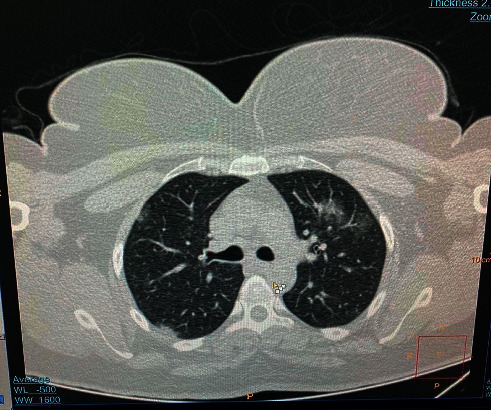
Patchy ground-glass and peripheral infiltration in chest CT scan.

A 35-year-old woman presented with anosmia after recovery from COVID-19. About two weeks before the anosmia, she had experienced low-grade fever, dry coughing, and headache. Due to the outbreak of COVID-19, physicians evaluated her for SARS-CoV-2 paraclinical examinations. Her lab test revealed positive CRP (3+) and lymphopenia. Chest CT scan showed patchy ground-glass and peripheral infiltration (Figure 1). A throat swab sample was taken, and reverse real-time PCR assay confirmed SARS-CoV-2 infection.

She was referred to an otolaryngologist for her anosmia. A complete head and neck examination was performed. The nasal cavity, oral cavity, and cranial nerve functions (II–V, VII–IX, and XII) were normal. The patient was diagnosed with postviral olfactory dysfunction. Rhinocort spray, one puff BID for 10 days, was prescribed for her. However, no improvement was observed. A throat swab sample was taken, and reverse real-time PCR was performed again. The second PCR result was negative. Oral prednisolone was prescribed. After 6 days of consuming prednisolone, her anosmia reversed.

## 3. Discussion

The novel coronavirus SARS-CoV-2 is the seventh member of the Coronaviridae family, a positive-sense RNA virus, known to infect humans. The clinical features of patients confirmed to be infected with SARS-CoV-2 included lower respiratory tract illness with fever, dry cough, and dyspnoea [1].

In our early encounter with the COVID-19, before the pandemic and during the early days of dealing with this novel viral disease in our country, we found a potential relation that COVID-19 might have with anosmia in some of our patients.

Olfactory dysfunction is classified into conductive (physical blockage of airflow to olfactory mucosa) or sensorineural types (disruption of the olfactory neural signaling pathway) [2].

URI is the most common cause of olfactory dysfunction (sensorineural type). Human rhinovirus, picornavirus, parainfluenza virus type 2, human coronavirus, and Epstein–Barr virus have all been observed to cause postviral olfactory dysfunction. Olfactory dysfunction is frequently seen after a severe or prolonged course of common cold in female patients over 40 years of age. The virus might damage the olfactory epithelium and change the number and function of its receptors [3].

In the case of SARS-CoV-2, newly emerged literature studies suggest that the frequency of central nervous system symptoms due to SARS-CoV-2 is much lower than that of olfactory symptoms. It is a hypothesis that the olfactory disorders are not related to definitive viral damage to the neuronal cells. Nonneuronal cells that express ACE2 receptors such as the olfactory epithelium sustentacular cells, microvillar cells, Bowman's gland cells, horizontal basal cells, and olfactory bulb pericytes may be the target of the virus [4]. In the case of prolonged anosmia and long-lasting olfactory dysfunctions, the involvement of stem cells (which express lower levels of ACE2 receptors) could be cited as the cause. Yet, the exact pathogenic mechanism underlying the chemosensitive disorders in COVID-19 patients has not been elucidated. Furthermore, no treatment has been reported yet.

As for anosmia due to SARS-CoV-2, recent case series showed a high rate of recovery of olfactory function within 1-2 weeks after the onset of the dysfunction [5–7], although, in our patient, anosmia did not recover after 2 weeks of its onset. The sensorineural type olfactory dysfunction prognosis is poor and sometimes is irreversible. Postviral olfactory dysfunction shows a poor response to treatment and, if recovered, the majority of patients show improvement within 6 months. The sensorineural type olfactory dysfunction may not show an effective response to topical or oral steroids [8].

Interestingly, in our first patient, oral prednisolone therapy showed a good response, and the patient was treated after a short course of therapy. We observed the same positive response to oral prednisolone in our subsequent patients. We recommend treatment with oral prednisolone for patients with olfactory dysfunction after their PCR swab test becomes negative.

However, more studies are needed to validate our finding and to find other specific therapies to avoid long-lasting chemosensitive disorders.

## References

[1] Shi H., Han X., Jiang N. (2020). Radiological findings from 81 patients with COVID-19 pneumonia in Wuhan, China: a descriptive study. *The Lancet Infectious Diseases*.

[2] Cho S. H. (2014). Clinical diagnosis and treatment of olfactory dysfunction. *Hanyang Medical Reviews*.

[3] Suzuki M., Saito K., Min W.-P. (2007). Identification of viruses in patients with postviral olfactory dysfunction. *The Laryngoscope*.

[4] Sungnak W., Huang N., Huang N. (2020). SARS-CoV-2 entry factors are highly expressed in nasal epithelial cells together with innate immune genes. *Nature Medicine*.

[5] Lechien J. R., Chiesa-Estomba C. M., De Siati D. R. (2020). Olfactory and gustatory dysfunctions as a clinical presentation of mild-to-moderate forms of the coronavirus disease (COVID-19): a multicenter European study. *European Archives of Oto-Rhino-Laryngology*.

[6] Yan C. H., Faraji F., Prajapati D. P., Boone C. E., DeConde A. S. (2020). Association of chemosensoty dysfunction and COVID-19 in patients presenting with influenza-like symptoms. *International Forum of Allergy & Rhinology*.

[7] Vaira L. A., Deiana G., Fois A. G. (2020). Objective evaluation of anosmia and ageusia in COVID-19 patients: a single-center experience on 72 cases. *Head and Neck*.

[8] Hummel T. (2000). Perspectives in olfactory loss following viral infections of the upper respiratory tract. *Archives of Otolaryngology-Head & Neck Surgery*.

